# MSERNet: a multi-scale edge-guided network for JPEG compression artifact restoration in retrospective ovarian ultrasound images

**DOI:** 10.3389/frai.2026.1964967

**Published:** 2026-10-30

**Authors:** K. Komala, S. Kalaivani

**Affiliations:** School of Computer Science Engineering and Information Systems, Vellore Institute of Technology, Vellore, Tamil Nadu, India

**Keywords:** DICOM, image restoration, JPEG compression, multi-scale feature extraction, ovarian ultrasound, deep learning, residual learning, transfer learning

## Abstract

**Introduction:**

JPEG compression is widely used to store and transmit medical ultrasound images but can introduce artifacts that affect image quality and subsequent image analysis. This study proposes a Multi-Scale Edge Guided Restoration Network (MSERNet) for reducing JPEG compression artifacts while preserving tissue textures and anatomical boundaries in ultrasound images.

**Methods:**

An ethically approved, anonymized private ovarian ultrasound dataset was curated and used to generate JPEG-compressed and recompressed images at multiple quality levels. MSERNet integrates multi-scale feature extraction, adaptive feature fusion, and edge refinement within a residual learning framework. The model was evaluated on the private dataset and an independent public ultrasound dataset using the Structural Similarity Index (SSIM), Peak Signal-to-Noise Ratio (PSNR), and Visual Information Fidelity (VIF). Comparative experiments with existing restoration methods, ablation analysis, repeated-run evaluation, statistical analysis, and computational analysis were performed. Cross-dataset evaluation assessed generalization without additional fine-tuning. A downstream binary classification experiment using a transfer learning-based ResNet50 classifier was conducted on restored images, with performance assessed using accuracy, precision, sensitivity, specificity, F1-score, and area under the curve (AUC).

**Results:**

MSERNet reduced JPEG compression artifacts and improved image quality across the evaluated compression levels and datasets, with quantitative metrics supported by visual assessment. Comparative and ablation experiments assessed the method’s performance and component contributions, while repeated-run evaluation examined consistency. Cross-dataset evaluation demonstrated stable restoration performance without additional fine-tuning. The downstream classification experiment indicated that the restored images retained information relevant to cystic-versus-normal classification.

**Discussion:**

These findings highlight the potential of MSERNet to improve the quality of JPEG-compressed ultrasound images for subsequent analysis. Further validation on larger and more diverse datasets is needed to assess its generalizability and applicability to other medical image-analysis tasks.

## 1 Introduction

At present, ultrasound imaging is widely applied in routine clinical practice because of its real-time imaging ability, portability, safety, and low cost compared with other imaging methods, such as computed tomography (CT) and magnetic resonance imaging (MRI) (Szabo, 2014). In contemporary health care processes, compression techniques are often employed to store, transmit, and archive ultrasound images with minimum storage space and communication bandwidth. While the irreversible compression is acceptable in medical imaging systems for diagnostic purposes, excessive compression can cause artifacts like blocking distortion, blurring and loss of speckle texture, which can impact the visual interpretation and the performance of automated image analysis (Clunie, 2000). There has been a recent surge in the use of deep learning techniques, specifically Convolutional Neural Networks (CNN) and Transformer-based models, to minimize Joint Photographic Expert Group (JPEG) artifacts under different compression settings (Li et al., 2024; Liang et al., 2021). Although some recent advances have been made, few studies have investigated the compression-aware restoration on clinically realistic datasets with well-controlled degradation strategies. Furthermore, various problems are still not well resolved in ultrasound restoration, including consistent preprocessing, photometric standardization, and reliable lossless ground-truth data. This study proposes a clinically relevant approach to modeling and reconstruction of compression artifacts in ovarian ultrasound images from raw Digital Imaging and Communications in Medicine (DICOM) data. The approach involves a common pre-processing pipeline to obtain images of uniform quality and regions of interest extracted properly, and simulated multi-level and recompression JPEG images under controlled conditions to mimic real clinical conditions. Moreover, a residual CNN is proposed to effectively remove compression artifacts while maintaining important speckle patterns and anatomical boundaries.

## 2 Related works

Medical image reconstruction is a key technique for the precise analysis and diagnosis. Previous research primarily has been concerned with the problem of noise reduction and the minimization of visible artifacts, such as adaptive filtering and median smoothing. In many situations these methods could be used to enhance image quality while retaining key information. Histogram equalization was another commonly used technique to enhance contrast. Although simple, such methods are not always reliable in more complex cases. Their performance is limited because they depend on fixed assumptions, which may not reflect real clinical data (Ahemed et al., 2025). With the increasing use of digital storage, image compression has become more common in clinical practice. However, maintaining image quality during compression remains a challenge. Lossy compression can introduce visible artifacts and remove fine image details. In addition, many existing studies use relatively small datasets or synthetically generated images, which may not fully represent the characteristics of real clinical data.

There are various image compression methods that are used to save space and speed up the transfer process, but they can introduce visual artifacts that reduce the image quality. The Discrete Cosine Transform (DCT) and Discrete Wavelet Transform (DWT) are two classical transforms that are often used for compression. These methods were investigated in Chlubna and Zemcik (2025) and it was pointed out that quantization causes the loss of high-frequency details which leads to the occurrence of blocking artifacts and ringing artifacts. Also, Kulkarni and Dixit (2025) showed better compression efficiency. But it did not concentrate on repair of artifacts. To achieve better compression quality, some hybrid methods are developed by combining DCT and Singular Value Decomposition (SVD). Despite, this study examines the existing restoration techniques. It also contrasts their advantages and disadvantages in various applications. In the era of deep learning, image restoration has undergone tremendous changes. The author (Pai et al., 2026) gave a detailed review of CNN, Generative Adversarial Network (GAN), and transformer-based restoration models. The results demonstrated significant enhancements in tasks like denoising, deblurring, and super-resolution. The paper (El-Shafai et al., 2023) developed into the application of deep learning for super-resolution in medical images. Moreover, (Yaqub et al., 2022) explored the reconstruction techniques for various imaging modalities. These methods enhance the visual quality. But they do not explicitly discuss compression-related problems that commonly occur in practice. Restoration techniques are primarily aimed at noise reduction and enhancement of particular imaging modalities in medical imaging. Wavelet based method for ultrasound de-speckling and compression was presented in the study (Gupta et al., 2005). But this is a statistical modeling approach, not deep learning. In recent work, for example, Zhao and Li (2024) introduced an efficient ultrasound compression by means of frequency domain decomposition. But it did not focus on restoring artifacts. Modern deep learning models have achieved strong performance in medical image restoration. These include CNN-transformer hybrid models proposed by Jain et al. (2026), degradation-aware transformers by Wei et al. (2025), and RWKV-based architectures by Yang et al. (2026). However, these methods require high computational power. They also do not directly model compression artifacts, such as those caused by JPEG. The author (Zini et al., 2020) introduces deep residual autoencoder exploiting Residual-in-Residual Dense Blocks (RRDB) to remove artifacts in JPEG compressed images. The research (Lee et al., 2021) developed a lightweight densely connected network to reduce artifacts efficiently. Even though the results are promising, these methods are mostly trained on natural images. They are not well adapted to clinical data, especially ultrasound imaging. The author (Sivaanpu et al., 2026) highlighted that ultrasound image quality depends on several factors. These include technical aspects, patient conditions, and the operator’s skill. To measure restoration performance, different image quality assessment metrics have been developed. In Wang et al. (2004), the SSIM was introduced to measure perceptual similarity. In Sheikh and Bovik (2006), an information fidelity-based metric was proposed to evaluate visual quality. However, image references are typically required for these measures Additionally, existing image quality assessment methods may overlook clinically important structural information required for diagnosis. Although considerable progress has been made in image compression and restoration, these problems are often addressed independently, limiting their effectiveness in practical clinical workflows. Furthermore, advanced deep learning models require substantial computational resources and rarely consider multi-level JPEG compression, repeated recompression, or DICOM-specific degradation. Many studies are also evaluated using limited or synthetic datasets, with insufficient validation on heterogeneous clinical ultrasound images. Therefore, there remains a need for an effective restoration framework capable of preserving diagnostically important tissue textures and anatomical boundaries while maintaining robust performance across different compression levels and independent ultrasound datasets.

### 2.1 Research gap

Existing ultrasound image restoration methods rarely integrate multi-level JPEG compression, repeated recompression, and cross-dataset validation within a unified deep learning framework. In addition, limited attention has been given to restoring clinically acquired heterogeneous ultrasound images while preserving fine anatomical structures. These limitations may affect the reliability, practical use, and generalization of existing restoration methods in real clinical settings.

### 2.2 Motivation

Based on these limitations, this study introduces MSERNet, a residual network for restoring compressed ultrasound images. The proposed network uses multi-scale feature extraction, adaptive feature fusion, and edge refinement to reduce JPEG compression artifacts while preserving tissue texture and anatomical boundaries. The framework is evaluated using a private clinical ovarian ultrasound dataset and an independent public carotid ultrasound dataset. Additional cross-dataset experiments are performed at different JPEG compression and recompression levels to assess the performance and generalization of the proposed method.

### 2.3 Main contributions

- A Multi-Scale Edge-Guided Restoration Network (MSERNet) is proposed for JPEG-compressed ultrasound images. It uses multi-scale feature extraction, adaptive feature fusion, and edge refinement to reduce compression artifacts while preserving tissue texture and anatomical boundaries.
- A standardized preprocessing pipeline and controlled JPEG compression and recompression were applied to simulate realistic clinical imaging conditions.
- MSERNet was systematically evaluated using image-quality metrics, comparisons with traditional and deep-learning-based restoration methods, repeated training runs, cross-dataset evaluation, and downstream classification to assess restoration consistency and preservation of relevant image information.

## 3 Materials and methods

### 3.1 Overview of the proposed work

This proposed MSERNet, a deep learning for restoring JPEG compression artifacts in clinical retrospective ultrasound images. The framework improves image quality while preserving important anatomical structures. It is designed to handle images with different levels of JPEG compression. The overall workflow is shown in Figure 1. The process begins with the preparation of ultrasound images from both private and public datasets. The original images are converted into lossless PNG images to serve as the reference (ground truth). Multiple JPEG compression levels are generated from each reference image to create degraded input images. During training, the compressed images are used as inputs, while the corresponding PNG images are used as the target outputs. MSERNet learns to estimate the missing image details caused by JPEG compression. At inference, the trained model restores previously unseen compressed ultrasound images without requiring any additional processing. The performance of the proposed framework is evaluated using PSNR, SSIM, and VIF. The private and public datasets are evaluated separately and a cross-dataset validation is carried out by testing the private dataset directly on the public dataset without retraining the model. In this experiment, the generalization capability of the proposed framework is evaluated.

**Figure 1:**
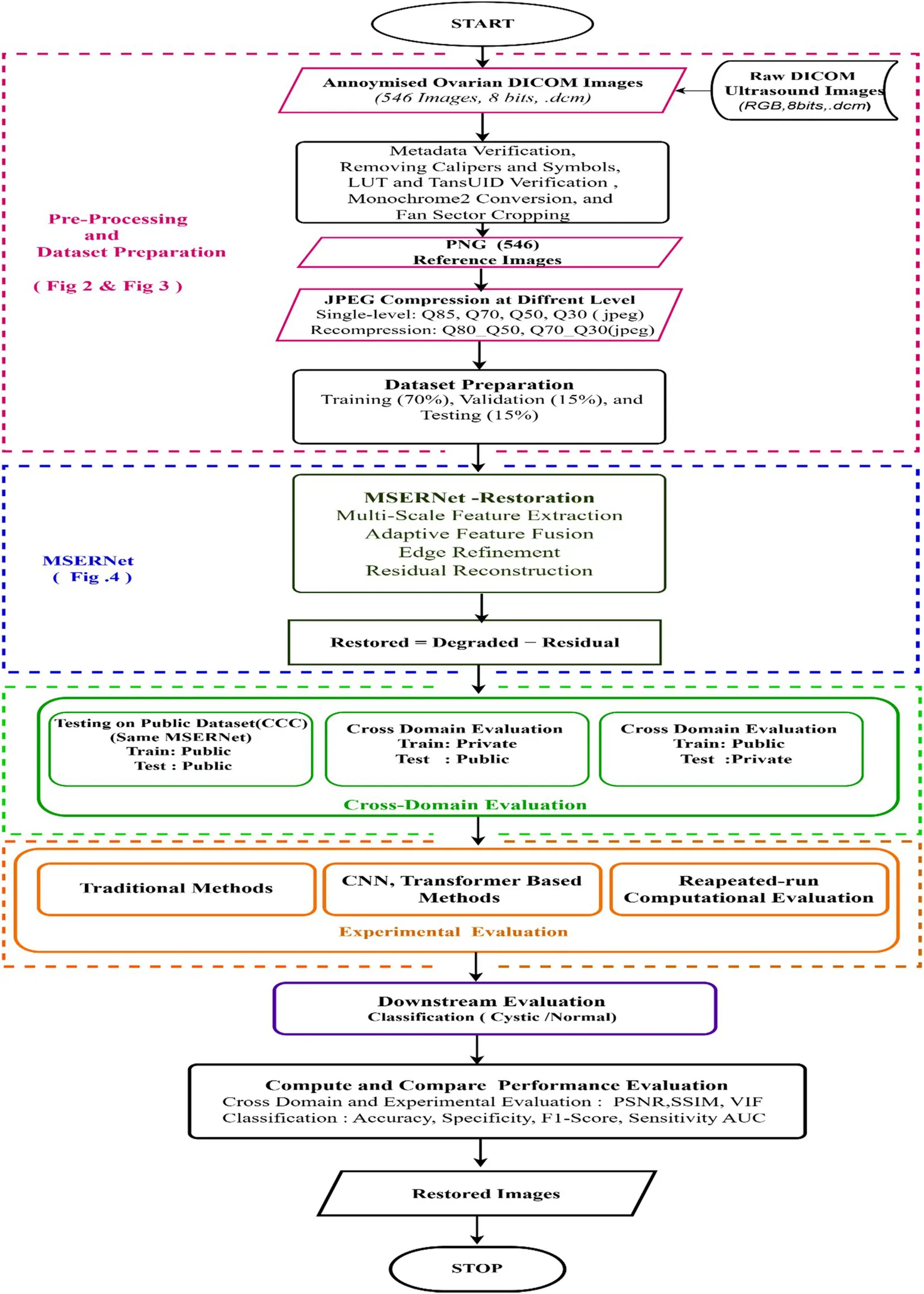
Flowchart: Overall work flow of MSERNet.

### 3.2 Dataset description

In this research work two sets of ultrasound images were used. The first data set was a retrospective, anonymized ovarian ultrasound dataset obtained from ES Hospital, India, from January to December 2024, during routine clinical examinations. The study was approved by the Institutional Ethics Committee of Vellore Institute of Technology (VIT), Vellore, India. The second dataset was the Common Carotid Artery (CCA) ultrasound dataset available from the Mendeley Data repository (Momot, 2022). Each dataset was pre-processed based on its original image format and acquisition characteristics. The reference and JPEG-compressed images were then generated for the restoration experiments. The main characteristics of both datasets are summarized in Table 1. The private dataset contained 546 anonymized ovarian ultrasound DICOM images. The images were obtained from separate patient-coded folders, with one image selected from each folder. The 546 images were divided into training, validation, and testing subsets using a stratified 70%/15%/15% split. Since each selected image came from a separate patient-coded folder, the same patient was not included in more than one subset.

**Table 1:** Private and public dataset details.

Characteristics	Private dataset	Public dataset (Momot, 2022)
Anatomy	Ovary	Common carotid artery
Imaging modality	Ultrasound	Ultrasound
Transducer type	Curved and linear	Linear probe
Original format	DICOM	PNG
De-identification	Yes	Yes
Patient/subject information	Patient-level organization	11 Subjects
Image dimension	2D	2D
Original resolution	600 × 800 × 3	709 × 749 × 3
Bit depth	8-bit	8-bit
Total Ultrasound Images Available	546	1,100
Images used in study	546	546 randomly selected
Minimum file size	36.73 KB	105.66 KB
Maximum file size	110.94 KB	332.55 KB
Average file size	77.84 KB	236.99 KB
Ethical approval/license	Institutional Ethics Committee for Human studies (IECH) -Vellore Institute of Technology, India	CC BY 4.0 (Mendeley Data)
Data collection period	January 2024 – December 2024	14-Nov-2022
After preprocessing – Image format	PNG	PNG
After preprocessing – Image resolution	468 × 650 × 1	468 × 650 × 1
After preprocessing – Images used	546	546
JPEG compression levels	Q30, Q50, Q70, Q85	Q30, Q50, Q70, Q85
Repeated compression	Q70 → Q30, Q85 → Q50	Q70 → Q30, Q85 → Q50

### 3.3 Preprocessing

The images from both datasets were pre-processed before model development. The aim was to create standardized reference images for the restoration framework. The different preprocessing steps were applied to the private and public datasets because their original image formats were different. The private ovarian ultrasound images were saved in DICOM format. All images were initially checked for complete anonymization. Before further processing patient identifiers and non-diagnostic annotations were removed. The images were then converted from RGB photometric interpretation to grayscale and saved as lossless PNG images. This conversion kept the original image quality and gave a reference for future analysis. The data from the public CCA (Momot, 2022) was available in PNG format. All the images were converted to gray scale and resized to the image resolution used in this study. This provided a constant size for the images when training and testing the model. After preprocessing, all reference images had a uniform resolution of 468 × 650 pixels and served as the reference image for the restoration framework. The entire preprocessing process is shown in Figure 2.

**Figure 2:**
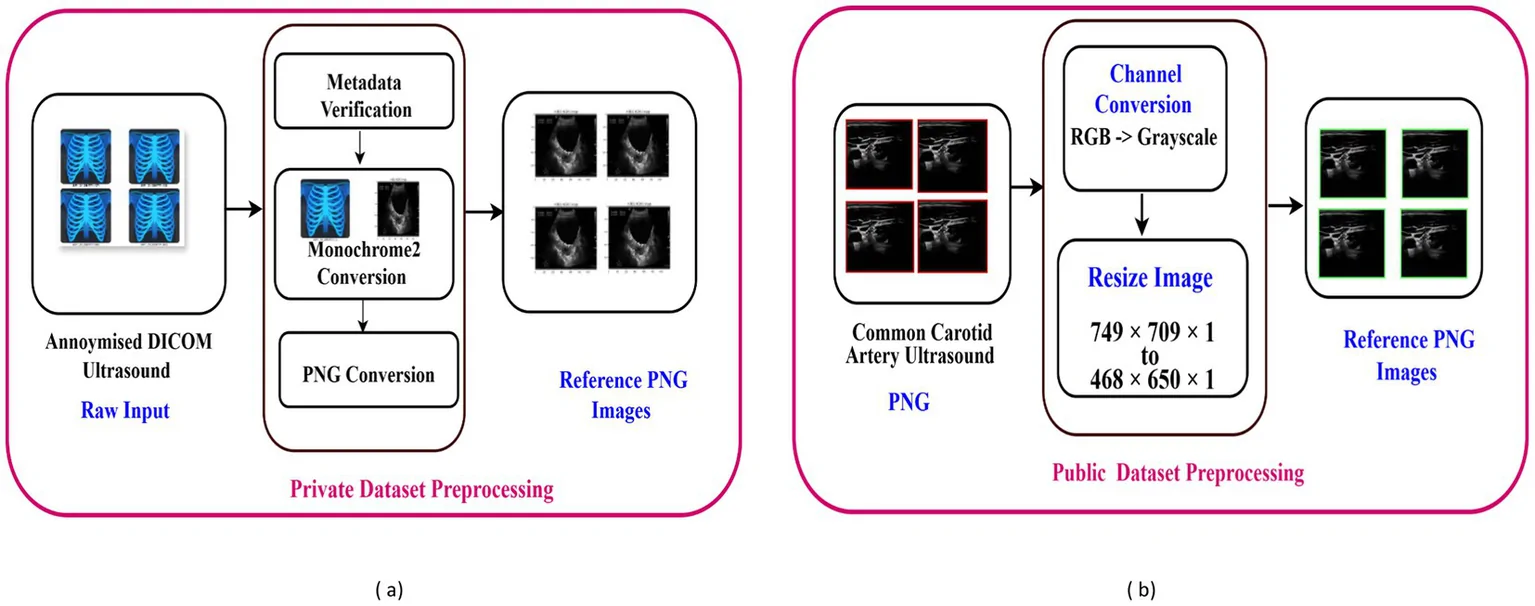
Preprocessing workflow: **(a)** Private ovarian ultrasound dataset and **(b)** Public CCA ultrasound dataset (Momot, 2022).

### 3.4 Multi-level JPEG compression dataset generation

The pre-processed PNG images were used as reference (ground-truth) images for generating JPEG-compressed images. PNG was chosen because it is a lossless image format that does not introduce compression artifacts, thereby preserving the original image quality. Before generating any JPEG-compressed or recompressed versions, the 546 source images were divided into mutually exclusive training, validation, and test subsets of 382, 82, and 82 images, respectively, using a fixed random seed of 42. As each source image corresponds to a different patient, this split was performed at the patient level. All degradation versions derived from the same source image were kept within the same subset, thereby preventing patient and source-image leakage between the training, validation, and test sets.

Each reference image was compressed at four JPEG quality factors (Q30, Q50, Q70, and Q85) to assess the restoration performance for various compression settings. The lower the quality factor, the more compression artifacts were visible and the higher the quality factor, the more image details were preserved. Two repeated compression scenarios (Q70_Q30 and Q85_Q50) were also generated to simulate images which had been compressed multiple times. These are real-life examples of medical images being compressed many times over during storage, transmission or archiving. The number of images in each compression level was the same as that in the corresponding reference dataset. The paired sets of data were JPEG-compressed input images and their corresponding PNG reference images. The image pairs were employed for training, validation, and quantitative evaluation of the model. The clean PNG, JPEG-compressed, and recompressed images were maintained in separate folders while retaining the same source-image identifier across the corresponding versions. The entire process of data generation is shown in Figure 3.

**Figure 3:**
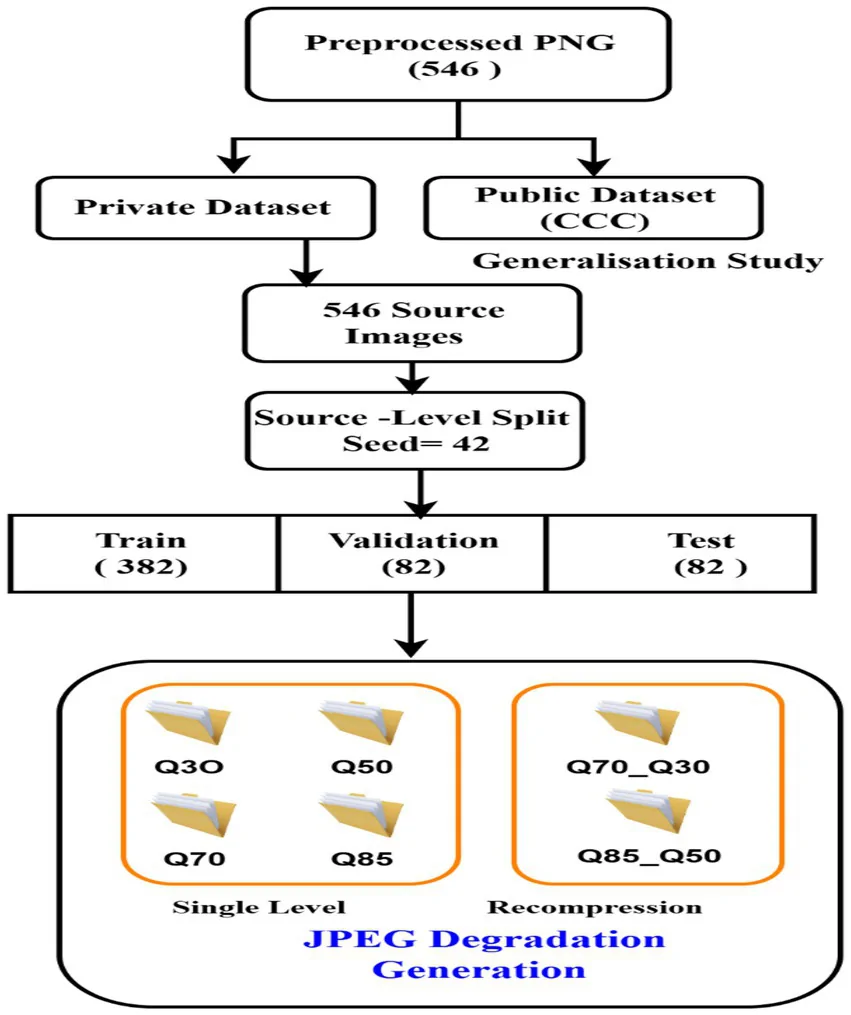
Multi-level JPEG compression dataset generation workflow.

### 3.5 Proposed MSERNet architecture

JPEG compression introduces artifacts that degrade fine textures, structural boundaries, and subtle anatomical details in ultrasound images. To address these challenges, the proposed Multi-Scale Edge-Guided Restoration Network (MSERNet) was designed as a restoration framework that combines multi-scale feature learning, adaptive feature fusion, edge refinement, and residual- based image restoration. Each module performs a specific task while collectively improving restoration quality. The overall architecture of the proposed MSERNet is illustrated in Figure 4. The network receives a JPEG-compressed grayscale ultrasound image as input. An Initial Feature Extraction Block, consisting of two consecutive 3 × 3 convolution layers with ReLU activation, extracts low-level image features for subsequent processing.

**Figure 4:**
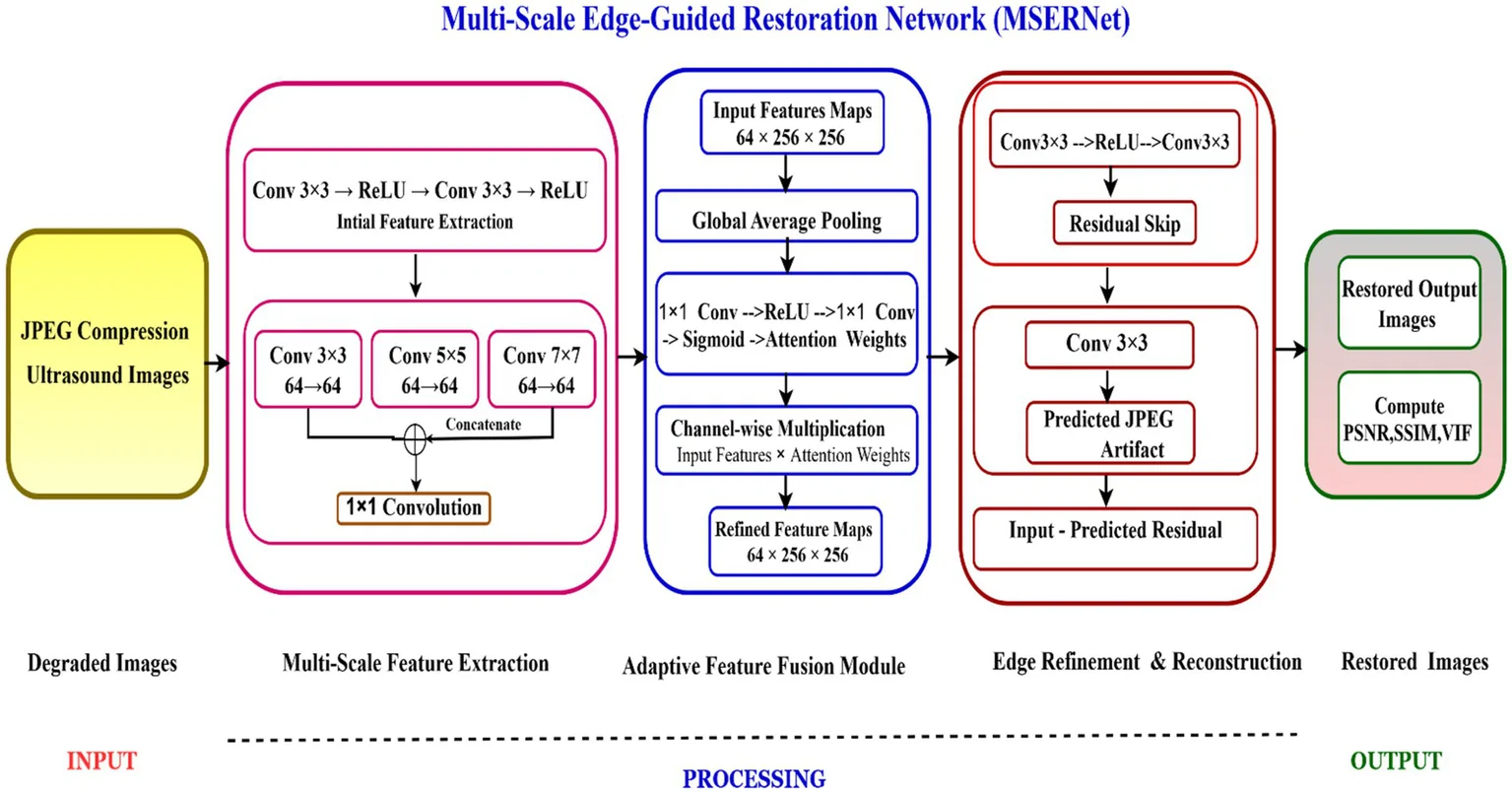
Proposed Architecture.

The extracted features are passed to the Multi-Scale Feature Extraction Block, where parallel 3 × 3, 5 × 5, and 7 × 7 convolution layers capture image information at different receptive fields. The resulting feature maps are concatenated and fused through a 1 × 1 convolution layer, producing a compact feature representation that preserves both fine textures and broader structural information. Next, the Adaptive Feature Fusion Module applies channel attention using global average pooling to emphasize informative features and suppress less relevant responses. This enables the network to emphasize informative feature responses associated with compression artifacts. The refined features are subsequently processed by the Edge Refinement Module, which employs two 3 × 3 convolution layers with a residual connection to enhance structural boundaries and preserve important anatomical details. Finally, a residual prediction layer estimates the compression-artifact residual. The restored image is obtained by subtracting the estimated residual from the compressed input image. This residual learning strategy allows the network to concentrate on artifact removal while preserving the original image content. The proposed MSERNet integrates four complementary modules within a single restoration framework: multi-scale feature extraction, adaptive feature fusion, residual edge refinement, and residual- based image restoration. Unlike conventional CNN-based restoration networks that primarily rely on sequential convolution, this integrated design enables more effective feature representation and artifact suppression while maintaining low computational complexity, making MSERNet suitable for ultrasound image restoration and cross-dataset generalization.

The key computational steps of the proposed MSERNet are summarized below using concise mathematical formulations.

In Equation 1, F denotes the input feature map and denotes the $F_{\mathrm{MS}}\;$ fused multi-scale feature representation used to capture features at different spatial scales.

$$F_{\mathrm{MS}}={\text{Conv}}_{\left(1\times 1\right)}(\text{Concat}[\text{ReLU}({\text{Conv}}_{\left(3\times 3\right)}(F)),\text{ReLU}({\text{Conv}}_{\left(5\times 5\right)}(F)),\text{ReLU}({\text{Conv}}_{\left(7\times 7\right)}(F))])$$ (1)

In Equation 2, A denotes the channel-attention weights, and $F_{A}$ denotes the adaptively weighted feature map used to emphasize informative feature responses.

$$A=\sigma (W_{2}\;\delta (W_{1}\;\mathrm{GAP}(F_{\mathrm{MS}}))),F_{A}=F_{\mathrm{MS}}⊙A$$ (2)

Where GAP denotes global average pooling W_1_ and W_2_ denote learnable convolution transformations, δ denotes ReLU activation, σ denotes the sigmoid function, and ⊙ denotes element-wise multiplication.

In Equation 3, $F_{E}\;$ denotes the refined feature map, and $\mathrm{Con}v_{3\times 3}^{\left(1\right)}$ and $\mathrm{Con}v_{3\times 3}^{\left(2\right)}\;$ denotes the two successive 3×3 convolution layers used to preserve local structures and anatomical boundaries.

$$F_{E}=F_{A}+{\text{Conv}}_{\left(3\times 3\right)}^{\left(2\right)}\;(\text{ReLU}({\text{Conv}}_{\left(3\times 3\right)}^{\left(1\right)}(F_{A})))$$ (3)

In Equation 4, $R_{D}$ denotes the estimated compression-artifact residual, $\;I_{D}$ denotes the JPEG-compressed input image, and $\;I_{R}\;\;\;$ denotes the restored image used to reconstruct the artifact-reduced image.

$$R_{D}={\text{Conv}}_{\left(3\times 3\right)}(F_{E}),I_{R}=I_{D}-R_{D}\;$$ (4)

In Equation 5, ${ℒ}_{1}$ denotes the L1 loss, N denotes the number of training samples, $I_{R}^{\left(i\right)}$ denotes the restored image for the i -th sample, $I_{\mathrm{GT}}^{\left(i\right)}$ denotes the corresponding ground-truth image used to measure reconstruction error during training.

$$L_{1}=(\frac{1}{N})\sum_{i=1}^{N}|I_{R}^{\left(i\right)}-I_{\mathrm{GT}}^{\left(i\right)}|$$ (5)

Algorithm 1 Overall workflow of the proposed MSERNet framework.

**Input:**

Private ovarian ultrasound DICOM dataset
Public carotid ultrasound dataset (Momot, 2022)

**Output:**

Restored ultrasound images with reduced JPEG compression artifacts

**Step 1**: Start

**Step 2**: Dataset Preparation and Preprocessing

2.1. Preprocessing

*Private Dataset*

- Collect anonymized ovarian ultrasound DICOM images with ethical approval.
- Perform Deidentification.
- Verify and remove the Lookup Table (LUT), if present.
- Convert DICOM images to MONOCHROME2 grayscale.
- Save the processed images as lossless PNG ground-truth images.
  *Public Dataset*
- Randomly select 546 images from the public carotid ultrasound dataset (Momot, 2022).
- Resize all images to match the spatial resolution of the private dataset.
- Convert RGB (3-channel) images to grayscale (1-channel).

2.2. Compression and Dataset Preparation

- Generate JPEG-compressed images at quality levels Q30, Q50, Q70, and Q85.
- Generate recompressed images at Q70_Q30 and Q85_Q50.
- Create paired datasets consisting of compressed images and corresponding PNG ground-truth images.
- Split the datasets into training (70%), validation (15%), and independent test sets (15%).

**Step 3: MSERNet Restoration Architecture**

- Input the compressed ultrasound image into MSERNet.
- Perform multi-scale feature extraction.
- Fuse multi-scale features using adaptive feature fusion.
- Refine anatomical edges using the edge refinement module.
- Reconstruct the restored ultrasound image using residual learning.

**Step 4: MSERNet Training and Cross Evaluation**

- Private Dataset → Train MSERNet → Test on Private
- Public Dataset → Train MSERNet → Test on Public
- Cross-Domain → Train on Private → Test on Public
- Cross Domain → Train on Public → Test on Private
- Evaluate restoration using PSNR, SSIM, and VIF.

**Step 5: Model Analysis**

- Compare MSERNet with existing restoration methods.
- Perform component-wise ablation analysis.
- Perform repeated-run evaluation using different random seeds.
- Measure parameters, model size, MACs, FLOPs, inference time, and GPU memory usage.

**Step 6: Statistical Validation**

- Perform a paired t-test on the independent test images.
- Evaluate statistical significance at α = 0.05.
- Paired t-test comparison of restoration performance between the public and cross-domain datasets.

**Step 7: Downstream Classification**

- Apply the trained ResNet50 classifier to MSERNet-restored images.
- Evaluate accuracy, precision, sensitivity, specificity, F1-score, and AUC.
- Analyze the corresponding confusion matrices.

**Step 8: Output**

- Generate restored ultrasound images with reduced JPEG compression artifacts.
- Report quantitative, statistical, computational, and downstream classification results.

### 3.6 Training strategy

The proposed MSERNet was implemented in PyTorch and trained on an NVIDIA Tesla T4 GPU (CUDA 12.8). For the private dataset, the 546 source images were divided into 70% training, 15% validation, and 15% testing, corresponding to 382, 82, and 82 images, respectively, as shown in Table 2. The source-level split was performed before generating the JPEG-compressed and recompressed versions, and all degradation variants of the same source image were retained within the same data split. Training was performed using 256 × 256 image patches with a batch size of 8. The network parameters were optimized using the Adam optimizer with an initial learning rate of 1 × 10^−4^, while the ReduceLROnPlateau scheduler adaptively adjusted the learning rate based on the validation loss. The model checkpoint with the lowest validation loss was selected as the final model for subsequent performance evaluation. The training and optimization settings are summarized in Table 3, while the experimental configurations for the private, public, and cross-domain evaluations are presented in Table 4. The public carotid ultrasound dataset contains a total of 2,200 files, comprising 1,100 ultrasound (US) images and 1,100 corresponding expert mask images. For the present study, only the 1,100 US images were considered. From these available US images, 546 images were randomly selected using a fixed random seed of 42 and stored separately. The selected images were subjected to the same preprocessing, JPEG compression, recompression, and experimental procedures as the private dataset. The selection of 546 images was made to maintain a comparable dataset size between the public and private datasets.

**Table 2:** Dataset split used for training and evaluation.

Dataset	Total images	Training	Validation	Test
Private dataset	546	382	82	82
Public dataset (CCC)	546	382	82	82

**Table 3:** Common training configuration of the proposed MSERNet.

Parameter	Value
Framework	PyTorch 2.11.0
Hardware	NVIDIA Tesla T4 GPU (CUDA 12.8)
Dataset split	70% Training, 15% Validation, 15% Testing
Batch size	8
Patch size	256 × 256
Training epochs	100
Data augmentation	None
Random seed	42
Optimizer	Adam
Initial learning rate	1 × 10^−4^
Learning rate scheduler	ReduceLROnPlateau
Loss function	L1 Loss
Trainable parameters	466,641 (0.467 M)

**Table 4:** Experimental settings for the proposed MSERNet.

Experiment type	Training	Testing	Evaluation strategy
Private	Private	Private	Evaluation of MSERNet on the private dataset
Public	Public	Public	Evaluation of the same MSERNet architecture on the public dataset
Cross-domain (Private → Public)	Private	Public	Evaluation of cross-domain generalization
Cross-domain (Public → Private)	Public	Private	Evaluation of cross-domain generalization

### 3.7 Performance evaluation

The restoration performance of the proposed MSERNet was evaluated using three commonly used image quality metrics PSNR, SSIM, and VIF. PSNR measures the pixel-level similarity between the restored and reference images. SSIM evaluates structural similarity based on luminance, contrast, and structural information (Wang et al., 2004). VIF measures the amount of visual information preserved after restoration (Sheikh and Bovik, 2006). Higher values indicate better restoration quality for all three metrics. The restored images were compared with their corresponding lossless PNG reference images for both the private and public ultrasound datasets.

### 3.8 Model training and cross-domain evaluation

Figure 5 shows the training and validation loss curves of MSERNet for the private and public ultrasound datasets. In both datasets, the losses gradually decreased during training, showing stable convergence. No clear signs of overfitting were observed. For the private ovarian ultrasound dataset (Figure 5a), the lowest validation loss was 0.005926 at epoch 14. The training and validation curves remained close to each other, indicating consistent model performance during training. For the public ultrasound dataset (Figure 5b), a similar pattern was observed. The lowest validation loss was 0.003119 at epoch 37. The lower validation loss indicates effective feature learning and stable optimization. Overall, the consistent convergence on both datasets demonstrates the reliability of the proposed training strategy and supports the restoration results presented in the following sections. Table 4 summarizes the four experimental settings designed to evaluate the proposed MSERNet on the private dataset, public dataset, and two cross-domain evaluation scenarios.

**Figure 5:**
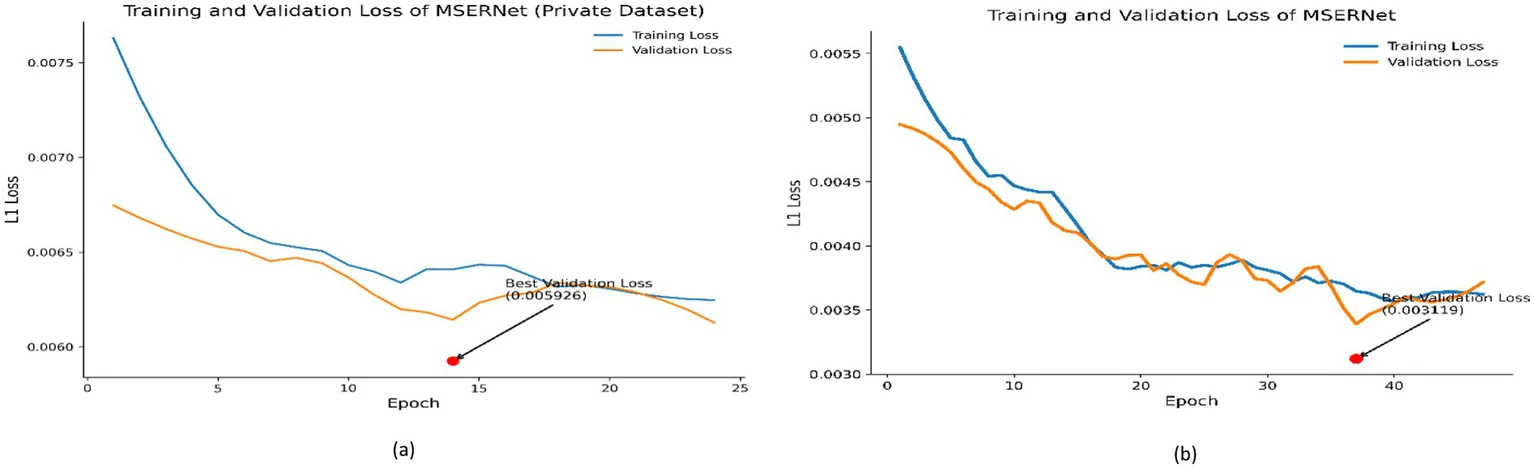
Training and validation loss curves of MSERNet: **(a)** Private dataset and **(b)** Public dataset.

### 3.9 Experimental evaluation

To evaluate the effectiveness and robustness of MSERNet, comparative, ablation, repeated-run, computational, and downstream evaluations were conducted using consistent dataset splits, JPEG degradation settings, and evaluation metrics. The comparative evaluation covered conventional, deep CNN-based, recent CNN-based, and Transformer-based restoration methods. In addition, ablation experiments were performed to examine the contribution of the main MSERNet components, while five repeated runs were conducted to assess the stability of the results. Computational evaluation was performed to assess model parameters, computational complexity, inference time, and GPU memory usage. A downstream transfer-learning classification experiment was also conducted to assess whether the restored images retained information relevant to cystic-versus-normal classification.

#### 3.9.1 Comparative evaluation with existing restoration methods

To address the comparison with state-of-the-art restoration methodologies, representative conventional, deep CNN-based, recent CNN-based, and Transformer-based methods were evaluated against MSERNet under the same JPEG degradation conditions and evaluation settings. The conventional methods included Wiener filtering, bilateral filtering, and total variation (TV). ARCNN (Dong et al., 2015) and RRDB (Zini et al., 2020) were included as established deep CNN-based restoration methods, while PALMSNet (Jain et al., 2026) was included as a recent CNN-based method. The DCT-domain spatial-frequential Transformer proposed by Ouyang and Chen (2024) was included as a recent Transformer-based JPEG artifact restoration method. All comparison methods were evaluated using the same degradation conditions and restoration metrics as MSERNet.

##### 3.9.1.1 Traditional restoration methods

Three conventional restoration methods, Wiener filtering, bilateral filtering, and total variation (TV), were selected based on the methods reported in the systematic review by Ahemed et al. (2025). These methods were applied to the same JPEG-degraded images used for MSERNet and evaluated using the same restoration metrics.

##### 3.9.1.2 Artifact reduction convolutional neural network

The artifact reduction convolutional neural network (ARCNN) proposed by Dong et al. (2015) was included as a learning-based comparison method. ARCNN was evaluated on the same JPEG-degraded images and under the same evaluation settings used for MSERNet.

##### 3.9.1.3 PALMSNet

The PALMSNet method proposed by Jain et al. (2026) was adapted as a learning-based baseline for comparison with MSERNet. The adapted implementation was trained and evaluated using the same dataset split, JPEG degradation settings, and evaluation metrics used for MSERNet to provide a consistent comparison.

##### 3.9.1.4 Residual-in-residual dense block

The Residual-in-Residual Dense Block (RRDB) architecture was adapted from the JPEG restoration approach reported by Zini et al. (2020) and used as a deep-learning baseline for comparison with MSERNet. The adapted model consisted of five RRDB blocks with 64 feature channels and 16 growth channels and was trained using L1 loss and the Adam optimizer with a learning rate of 1 × 10^−4^.

##### 3.9.1.5 DCT-based transformer

The DCT-domain spatial-frequential Transformer proposed (Ouyang and Chen, 2024) was adapted from the authors’ implementation and evaluated as a representative Transformer-based JPEG artifact restoration baseline for comparison with MSERNet.

#### 3.9.2 Repeated-run evaluation

To assess the stability of MSERNet, the training process was repeated five times using different training seeds (42, 123, 2024, 7, and 99). The dataset split was kept fixed across all runs, with 382 images or training, 82 for validation, and 82 for testing, using split seed 42. The results from the five runs were summarized using mean ± standard deviation.

#### 3.9.3 Computational evaluation

The computational characteristics of MSERNet were evaluated using the input resolution, total and trainable parameters, model size, multiply-accumulate operations (MACs), floating-point operations (FLOPs), inference time, and peak GPU memory usage. Inference measurements were obtained using an NVIDIA Tesla T4 GPU under the same evaluation conditions.

#### 3.9.4 Ablation evaluation

Four ablation variants were evaluated by individually removing multi-scale processing, channel attention, edge refinement, or residual learning while keeping the remaining components and training settings unchanged. All variants used a 256 × 256 patch size, batch size of 8, 382 training images, 82 validation images, and a maximum of 100 epochs with early stopping. The experiments were performed using run seed 42 on an NVIDIA Tesla T4 GPU. Table 5 presents the ablation variants of MSERNet used to evaluate the contribution of the individual network components.

**Table 5:** Ablation variants of MSERNet used to evaluate the contribution of individual network components.

Ablation variant	Multi-scale	Channel attention	Edge refinement	Residual learning
Without multi-scale	×	√	√	√
Without channel attention	√	×	√	√
Without edge refinement	√	√	×	√
Without residual learning	√	√	√	×
Full MSERNet	√	√	√	√

#### 3.9.5 Downstream transfer-learning classification

A downstream binary classification task was performed to assess whether image restoration preserved information relevant to cystic-versus-normal classification. Transfer learning was selected because the labeled dataset was relatively small (352 images) for training a deep classifier from scratch. The 352 labeled images comprised 176 cystic and 176 normal images and were divided into 246 training, 53 validation, and 53 test images using a fixed 70:15:15 split with seed 42. A ResNet50 model pretrained on ImageNet was fine-tuned by replacing its original classification layer with a two-class fully connected layer. Cross-entropy loss and Adam optimization were used for training, with a learning rate of 1 × 10^−4^ and a batch size of 16. After training, the same classifier was applied to the complete set of 546 standardized images under clean, degraded, and MSERNet-restored conditions. Classification performance was evaluated using accuracy, sensitivity, specificity, precision, F1-score, and ROC-AUC, with the same 53-image test set maintained across the evaluated conditions. The experimental settings used for the downstream transfer-learning classification are summarized in Table 6.

**Table 6:** Experimental settings for downstream transfer-learning classification.

Parameter	Experimental setting
Downstream task	Binary classification: Cystic vs. Normal
Total Images	546 images
Labeled subset	352 images
Cystic/normal	176/176
Classifier	ResNet50 pretrained on ImageNet
Input size	224 × 224 pixels
Input channels	3
Training/validation/test	246/53/53
Split	70:15:15, fixed seed 42
Conditions evaluated	MSERNet-restored images
Classification layer	Two-class fully connected layer
Loss	Cross-Entropy
Optimizer	Adam
Learning rate	1 × 10^−4^
Batch size	16
Maximum epochs	30
Scheduler	ReduceLROnPlateau
Early stopping patience	7 epochs
Evaluation metrics	Accuracy, sensitivity, specificity, precision, F1-score, AUC
Hardware	NVIDIA Tesla T4

## 4 Results

The performance of MSERNet was evaluated using quantitative and visual analyses. Comparative, ablation, repeated-run, statistical, and computational analyses were performed to examine restoration performance and model behavior. Cross-dataset evaluation and downstream classification were conducted to assess the generalization of the model and the usefulness of the restored images for subsequent analysis. Visual comparisons were also presented to illustrate the changes in image quality after restoration.

### 4.1 Quantitative results on the private dataset

The proposed MSERNet was evaluated on the private ovarian ultrasound dataset under different JPEG compression levels. Table 7 summarizes the restoration results using PSNR, SSIM, and VIF. The highest restoration performance was obtained at Q85, with a PSNR of 47.06 ± 1.19 dB, SSIM of 0.9904 ± 0.0030, and VIF of 0.8026 ± 0.0551. At Q70, the restored images achieved a PSNR of 44.94 ± 1.00 dB, SSIM of 0.9646 ± 0.0058, and VIF of 0.7299 ± 0.0656. For Q50 and Q30, the model achieved PSNR values of 42.48 ± 1.15 dB and 39.33 ± 0.96 dB, respectively. The corresponding SSIM and VIF values are also reported in Table 7. Similar improvements were observed for the recompressed datasets (Q70_Q30 and Q85_Q50). Overall, MSERNet delivered consistent restoration performance across all compression levels.

**Table 7:** Restoration performance of MSERNet on the private ovarian ultrasound dataset (mean ± SD).

Compression type	JPEG Level	PSNR (dB)	SSIM	VIF
Single level	Q30	39.33 ± 0.96	0.9363 ± 0.0048	0.5667 ± 0.0713
	Q50	42.48 ± 1.15	0.9499 ± 0.0079	0.6569 ± 0.0704
	Q70	44.94 ± 1.00	0.9646 ± 0.0058	0.7299 ± 0.0656
	Q85	47.06 ± 1.19	0.9904 ± 0.0030	0.8026 ± 0.0551
Recompression	Q70_Q30	39.07 ± 0.96	0.9471 ± 0.0081	0.5527 ± 0.0723
	Q85_Q50	42.73 ± 1.02	0.9640 ± 0.0054	0.6603 ± 0.0667

### 4.2 Quantitative results on the public dataset

The same experiment was repeated on an independent public ultrasound dataset. Table 8 shows the results. Among the evaluated compression levels, Q85 produced the highest PSNR, SSIM, and VIF values (48.93 ± 1.28 dB, 0.9954 ± 0.0010, and 0.8916 ± 0.0121, respectively). At Q70, the model achieved a PSNR of 46.90 ± 1.23 dB, SSIM of 0.9923 ± 0.0016, and VIF of 0.8519 ± 0.0141. Q50 and Q30 also achieved high PSNR, SSIM, and VIF values after restoration. A similar trend was observed for the recompressed datasets (Q70_Q30 and Q85_Q50). These results indicate that MSERNet restored ultrasound images consistently under different JPEG compression settings.

**Table 8:** Restoration performance of MSERNet on the public ultrasound dataset (Momot, 2022) (mean ± SD).

Compression type	JPEG level	PSNR (dB)	SSIM	VIF
Single level	Q30	42.12 ± 1.20	0.9778 ± 0.0053	0.7236 ± 0.0194
	Q50	44.69 ± 1.19	0.9871 ± 0.0025	0.7985 ± 0.0164
	Q70	46.90 ± 1.23	0.9923 ± 0.0016	0.8519 ± 0.0141
	Q85	48.93 ± 1.28	0.9954 ± 0.0010	0.8916 ± 0.0121
Recompression	Q70_Q30	41.78 ± 1.19	0.9735 ± 0.0055	0.7113 ± 0.0198
	Q85_Q50	44.72 ± 1.19	0.9879 ± 0.0024	0.8013 ± 0.0157

### 4.3 Cross-domain evaluation

#### 4.3.1 Private (train) *vs* public (test)

The model trained on the private ovarian ultrasound dataset was directly tested on the public ultrasound dataset without further training or fine-tuning to assess its generalization ability. The results are shown in Table 9. The highest restoration performance was obtained at Q85, with a PSNR of 48.53 ± 1.21 dB, SSIM of 0.9950 ± 0.0010, and VIF of 0.8859 ± 0.0101. For Q70, the model achieved a PSNR of 46.05 ± 1.19 dB, SSIM of 0.9908 ± 0.0018, and VIF of 0.8343 ± 0.0129. The model also maintained consistent restoration performance for Q50, Q30, and the recompressed datasets (Q70_Q30 and Q85_Q50). These results demonstrate that the proposed MSERNet can effectively restore JPEG-compressed ultrasound images from an unseen dataset using a model trained only on the private dataset. Table 9 presents a comparison of performance metrics across the different datasets and compression levels, including the cross-domain (CD) evaluation.

**Table 9:** Cross-domain restoration results from the private to public ultrasound dataset (mean ± SD).

Compression type	JPEG level	PSNR (dB)	SSIM	VIF
Single level	Q30	40.86 ± 0.99	0.9159 ± 0.0092	0.7000 ± 0.0184
	Q50	43.09 ± 0.86	0.9299 ± 0.0118	0.7755 ± 0.0155
	Q70	46.05 ± 1.19	0.9908 ± 0.0018	0.8343 ± 0.0129
	Q85	48.53 ± 1.21	0.9950 ± 0.0010	0.8859 ± 0.0101
Recompression	Q70_Q30	40.61 ± 0.99	0.9143 ± 0.0088	0.6878 ± 0.0187
	Q85_Q50	43.04 ± 0.86	0.9286 ± 0.0120	0.7773 ± 0.0151

#### 4.3.2 Public (train) *vs* private (test)

To further assess the generalization capability of the proposed MSERNet, a reverse cross-domain experiment was conducted. In this setting, the model was trained on the public carotid ultrasound dataset and directly evaluated on the private ovarian ultrasound dataset without additional fine-tuning. This experiment was designed to examine the robustness of the proposed network when applied to an independent clinical dataset acquired from a different imaging source. The restoration performance was evaluated using PSNR, SSIM, and VIF across all JPEG compression and recompression levels as shown in Table 10. The results demonstrate that MSERNet maintains consistent restoration performance under cross domain conditions, indicating its ability to generalize across ultrasound datasets with different anatomical regions and acquisition characteristics.

**Table 10:** Cross-dataset evaluation of MSERNet (public-to-private) (mean ± SD).

Compression type	JPEG level	PSNR (dB)	SSIM	VIF
Single level	Q30	38.93 ± 1.04	0.9139 ± 0.0100	0.5665 ± 0.0768
	Q50	40.45 ± 1.17	0.9271 ± 0.0075	0.6469 ± 0.0775
	Q70	41.63 ± 1.25	0.9338 ± 0.0060	0.7050 ± 0.0755
	Q85	42.80 ± 1.38	0.9387 ± 0.0048	0.7518 ± 0.0725
Recompression	Q70_Q30	38.66 ± 1.04	0.9110 ± 0.0103	0.5525 ± 0.0775
	Q85_Q50	40.51 ± 1.14	0.9270 ± 0.0071	0.6524 ± 0.0743

### 4.4 Qualitative visual comparison

Figure 6 shows the reference PNG images from both datasets. Figure 7 shows the restoration results obtained using MSERNet under different JPEG compression levels and recompression settings. The compressed images exhibit blocking artifacts, blurred boundaries, and loss of fine tissue details, particularly at lower quality factors. After restoration, most of these artifacts are reduced while tissue structures and important image details are preserved. The restored images demonstrate clearer lesion boundaries and improved visual quality with minimal over-smoothing. Similar restoration performance is observed for both the private and public datasets, as well as during cross-dataset evaluation, indicating the robustness of the proposed model across datasets. These visual observations are consistent with the quantitative improvements reported in Table 11. Figure 8 presents line plots comparing the quantitative restoration performance of MSERNet across different JPEG compression levels for the private, public, and cross-dataset evaluation scenario.

**Figure 6:**
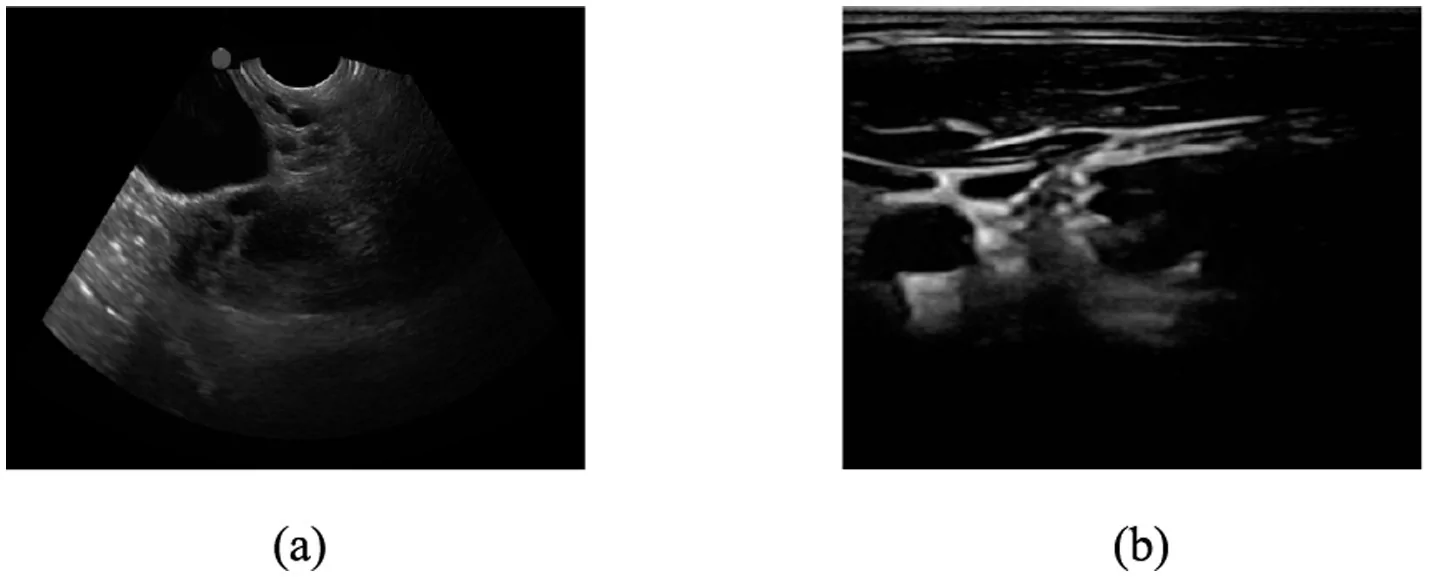
Reference PNG images: **(a)** Private dataset and **(b)** Public dataset (Momot, 2022).

**Figure 7:**
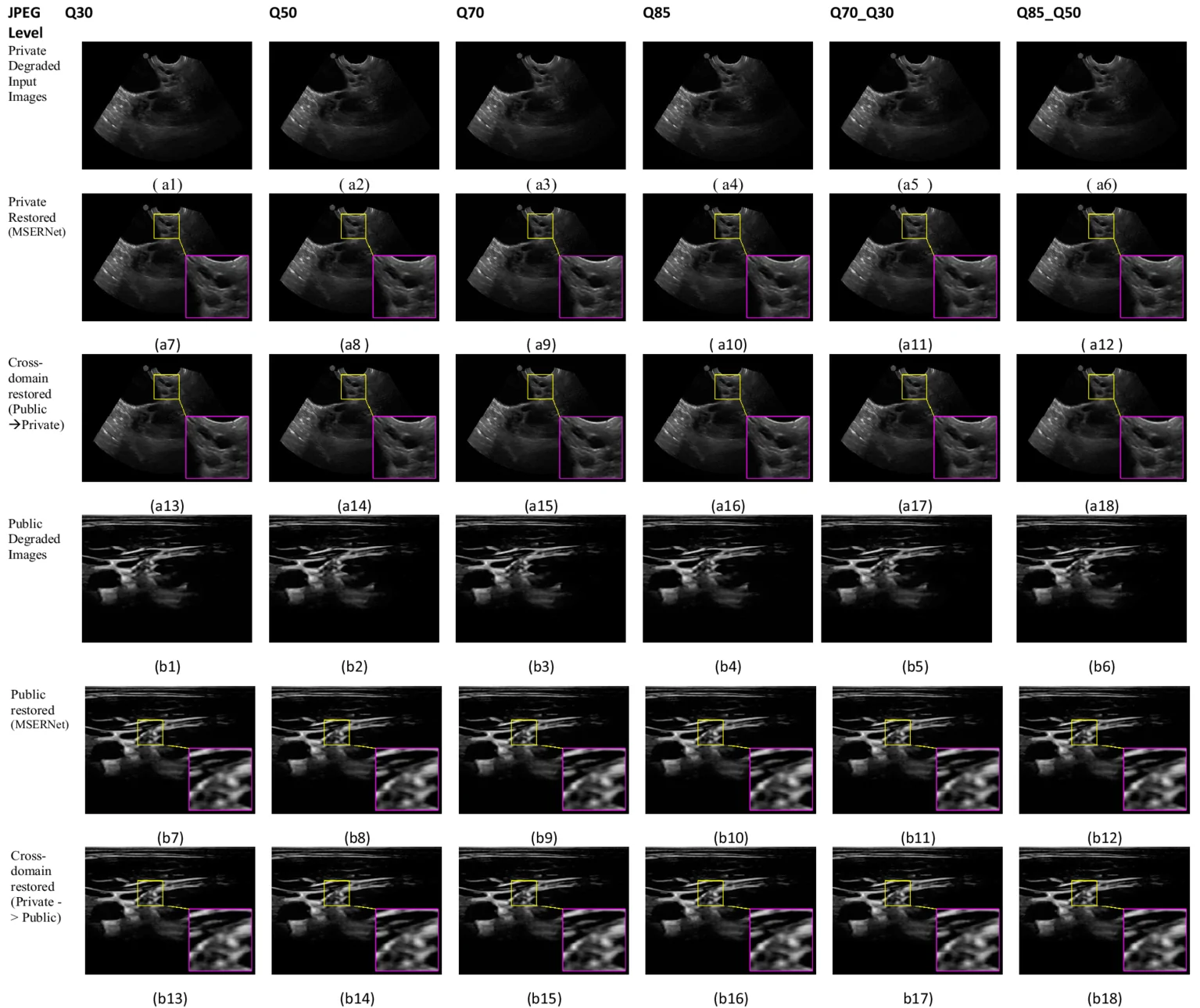
Qualitative comparison of MSERNet restoration results under different JPEG compression and recompression settings. The columns represent Q30, Q50, Q70, Q85, Q70_Q30, and Q85_Q50. For the private dataset, degraded input images are shown in (a1–a6), MSERNet-restored images in (a7–a12), and cross-domain restored images (Public→Private) in (a13–a18). For the public dataset, degraded input images are shown in (b1–b6), MSERNet-restored images in (b7–b12), and cross-domain restored images (Private→Public) in (b13–b18).

**Table 11:** Comparison analysis of performance metrics across different dataset and compression levels, including cross-domain (CD) evaluation.

JPEG levels	PSNR (Mean ± SD) ↑				SSIM (Mean ± SD) ↑				VIF (Mean ± SD) ↑			
	Private dataset	Public dataset	CD private–public	CD Public–Private	Private dataset	Public dataset	CD private–public	CD public–private	Private dataset	Public dataset	CD private–public	CD public–private
Q30	39.33 ± 0.96	42.12 ± 1.20	40.86 ± 0.99	38.93 ± 1.04	0.9363 ± 0.0048	0.9778 ± 0.0053	0.9159 ± 0.0092	0.9139 ± 0.0100	0.5667 ± 0.0713	0.7236 ± 0.0194	0.7000 ± 0.0184	0.5665 ± 0.0768
Q50	42.48 ± 1.15	44.69 ± 1.19	43.09 ± 0.86	40.45 ± 1.17	0.9499 ± 0.0079	0.9871 ± 0.0025	0.9299 ± 0.0118	0.9271 ± 0.0075	0.6569 ± 0.0704	0.7985 ± 0.0164	0.7755 ± 0.0155	0.6469 ± 0.0775
Q70	44.94 ± 1.00	46.90 ± 1.23	46.05 ± 1.19	41.63 ± 1.25	0.9646 ± 0.0058	0.9923 ± 0.0016	0.9908 ± 0.0018	0.9338 ± 0.0060	0.7299 ± 0.0656	0.8519 ± 0.0141	0.8343 ± 0.0129	0.7050 ± 0.0755
Q85	47.06 ± 1.19	48.93 ± 1.28	48.53 ± 1.21	42.80 ± 1.38	0.9904 ± 0.0030	0.9954 ± 0.0010	0.9950 ± 0.0010	0.9387 ± 0.0048	0.8026 ± 0.0551	0.8916 ± 0.0121	0.8859 ± 0.0101	0.7518 ± 0.0725
Q70_ Q30	39.07 ± 0.96	41.78 ± 1.19	40.61 ± 0.99	38.66 ± 1.04	0.9471 ± 0.0081	0.9735 ± 0.0055	0.9143 ± 0.0088	0.9110 ± 0.0103	0.5527 ± 0.0723	0.7113 ± 0.0198	0.6878 ± 0.0187	0.5525 ± 0.0775
Q85_ Q50	42.73 ± 1.02	44.72 ± 1.19	43.04 ± 0.86	40.51 ± 1.14	0.9640 ± 0.0054	0.9879 ± 0.0024	0.9286 ± 0.0120	0.9270 ± 0.0071	0.6603 ± 0.0667	0.8013 ± 0.0157	0.7773 ± 0.0151	0.6524 ± 0.0743

↑ symbol indicate higher value.

**Figure 8:**
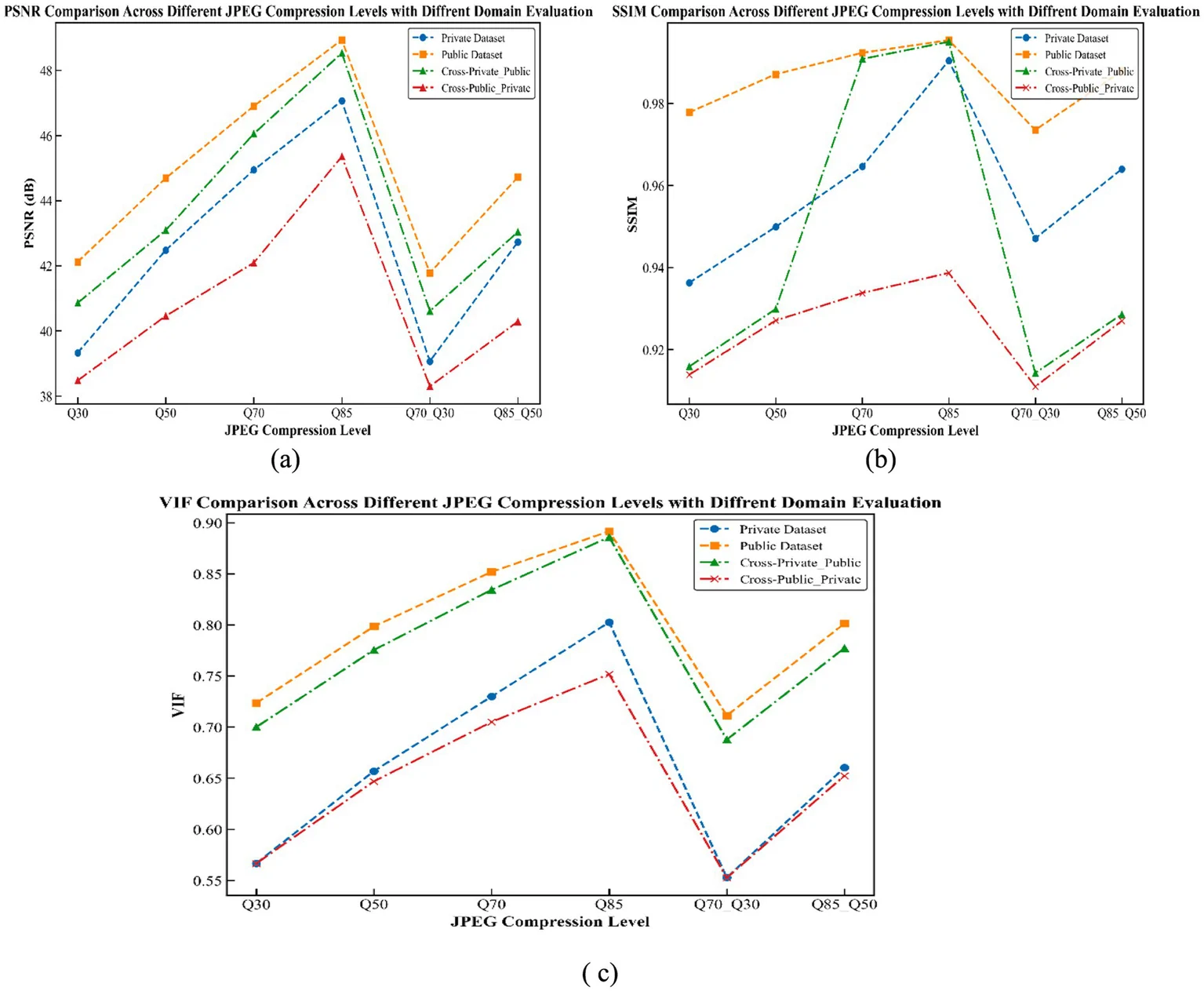
Performance comparison across JPEG compression levels **(a)** PSNR, **(b)** SSIM, and **(c)** VIF.

### 4.5 Comparison with existing restoration methods

The comparative results are presented in Table 12(a–c) as mean ± SD for PSNR, SSIM, and VIF. The restoration results varied across the evaluated methods and JPEG degradation levels. For PSNR, MSERNet obtained the highest values at Q50, Q70, and Q85, with 42.48 ± 1.15 dB, 44.94 ± 1.00 dB, and 47.06 ± 1.19 dB, respectively. The DCT Transformer (Ouyang and Chen, 2024) obtained higher PSNR at Q30 and Q70_Q30. For SSIM, MSERNet recorded the highest values at Q50 and Q85, with 0.9499 ± 0.0079 and 0.9904 ± 0.0030, respectively, while PALMSNet (Jain et al., 2026) recorded higher values under the other conditions. For VIF, MSERNet obtained the highest values at Q70 and Q85, with 0.7299 ± 0.0656 and 0.8026 ± 0.0551, respectively whereas Wiener filtering (Ahemed et al., 2025) obtained higher values under the remaining conditions. Overall, the highest value differed across methods depending on the degradation level and evaluation metric Table 12(a–c).

**Table 12:** Comparison of restoration performance of MSERNet and existing restoration methods under different JPEG degradation conditions, reported as mean ± standard deviation (SD) for (a) PSNR, (b) SSIM, and (c) VIF.

Methods	Traditional methods			Deep CNN methods		Recent CNN	Transformer	Proposed
JPEG Level	Total Variation (Ahemed et al., 2025)	Bilateral (Ahemed et al., 2025)	Wiener (Ahemed et al., 2025)	ARCNN (Dong et al., 2015)	RRDB (Zini et al., 2020)	PALMSNet (Jain et al., 2026)	DCT Transformer (Ouyang and Chen, 2024)	MSRENet
(a) Comparison of PSNR (Mean ±SD) ↑								
Q30	34.79 ± 0.89	36.08 ± 0.90	36.20 ± 1.09	38.73 ± 1.05	39.2310 ± 1.00	38.09 ± 1.18	**40.57 ± 1.03**	39.33 ± 0.96
Q50	34.95 ± 0.89	36.40 ± 0.90	37.13 ± 1.19	40.17 ± 1.19	41.0711 ± 1.13	39.35 ± 1.32	41.52 ± 1.03	**42.48 ± 1.15**
Q70	34.98 ± 0.87	36.52 ± 0.89	37.61 ± 1.19	40.67 ± 1.15	42.2955 ± 1.14	40.18 ± 1.41	42.35 ± 1.04	**44.94 ± 1.00**
Q85	35.17 ± 0.89	36.83 ± 0.91	38.55 ± 1.32	42.09 ± 1.38	44.4483 ± 1.36	38.09 ± 1.18	45.66 ± 0.86	**47.06 ± 1.19**
Q70_Q30	34.63 ± 0.89	35.88 ± 0.90	35.91 ± 1.08	38.53 ± 1.05	38.9860 ± 1.00	39.35 ± 1.32	**40.18 ± 1.01**	39.07 ± 0.96
Q85_Q50	34.98 ± 0.88	36.44 ± 0.90	37.18 ± 1.19	40.18 ± 1.18	41.0819 ± 1.11	40.18 ± 1.41	41.53 ± 1.01	**42.73 ± 1.02**
(b) Comparison of SSIM (Mean ±SD) ↑								
Q30	0.8875 ± 0.0160	0.9029 ± 0.0138	0.9338 ± 0.0115	0.9495 ± 0.0084	0.9501 ± 0.0080	**0.9511 ± 0.0111**	0.9375 ± 0.0085	0.9363 ± 0.0048
Q50	0.8900 ± 0.0165	0.8717 ± 0.0153	0.9070 ± 0.0133	0.9006 ± 0.0051	0.9368 ± 0.0049	0.9360 ± 0.0064	0.9409 ± 0.0063	**0.9499 ± 0.0079**
Q70	0.8522 ± 0.0178	0.9068 ± 0.0140	0.9208 ± 0.0114	0.9327 ± 0.0062	0.9646 ± 0.0059	**0.9650 ± 0.0077**	0.9548 ± 0.0045	0.9646 ± 0.0058
Q85	0.9016 ± 0.0177	0.9214 ± 0.0150	0.9573 ± 0.0132	0.9776 ± 0.0037	0.9898 ± 0.0032	0.9864 ± 0.0047	0.9760 ± 0.0027	**0.9904 ± 0.0030**
Q70_Q30	0.8842 ± 0.0166	0.8996 ± 0.0143	0.9302 ± 0.0117	0.9468 ± 0.0088	0.9475 ± 0.0083	**0.9491 ± 0.0116**	0.9350 ± 0.0138	0.9471 ± 0.0081
Q85_Q50	0.8899 ± 0.0164	0.9068 ± 0.0138	0.9409 ± 0.0113	0.9625 ± 0.0057	0.9643 ± 0.0055	**0.9653 ± 0.0074**	0.9526 ± 0.0132	0.9640 ± 0.0054
(c) Comparison of VIF (Mean ±SD) ↑								
Q30	0.5246 ± 0.0446	0.6531 ± 0.0386	**0.6668 ± 0.0235**	0.5626 ± 0.0728	0.5665 ± 0.0716	0.5608 ± 0.0715	0.6016 ± 0.0812	0.5667 ± 0.0713
Q50	0.5296 ± 0.0440	0.6639 ± 0.0381	**0.7180 ± 0.0215**	0.6452 ± 0.0740	0.6543 ± 0.0709	0.6534 ± 0.0709	0.6890 ± 0.0756	0.6569 ± 0.0704
Q70	0.5321 ± 0.0438	0.6713 ± 0.0385	0.7220 ± 0.0213	0.7069 ± 0.0729	**0.7229 ± 0.0665**	0.7183 ± 0.0667	0.7113 ± 0.0641	**0.7299 ± 0.0656**
Q85	0.5386 ± 0.0435	0.6832 ± 0.0391	0.7359 ± 0.0214	0.7599 ± 0.0686	0.7872 ± 0.0565	0.7800 ± 0.0570	0.7801 ± 0.0472	**0.8026 ± 0.0551**
Q70_Q30	0.5156 ± 0.0451	0.6410 ± 0.0394	**0.6810 ± 0.0251**	0.5498 ± 0.0739	0.5526 ± 0.0726	0.5546 ± 0.0725	0.5850 ± 0.0817	0.5527 ± 0.0723
Q85_Q50	0.5302 ± 0.0438	0.6657 ± 0.0376	**0.7159 ± 0.0212**	0.6493 ± 0.0704	0.6586 ± 0.0673	0.6578 ± 0.0677	0.6963 ± 0.0717	0.6603 ± 0.0667

Bold values indicate the best performance among the compared methods. ↑ indicates that higher values represent better performance.

### 4.6 Ablation studies

The ablation results are shown in Table 13. Each experiment removed one component from MSERNet and was compared with the complete model. Removing multi-scale processing, channel attention, residual learning, and edge refinement reduced PSNR by 0.01–1.99 dB, 0.53–2.59 dB, 1.01–3.20 dB, and 0.81–1.87 dB, respectively, across most degradation conditions. The complete MSERNet achieved the highest PSNR in five of the six conditions. For SSIM, the results varied across the degradation levels, with the complete model giving the highest values at Q50, Q70, and Q85. For VIF, the complete MSERNet gave the highest value at all six conditions. Removing multi-scale processing, channel attention, residual learning, and edge refinement reduced VIF by 0.0007–0.0194, 0.0112–0.0240, 0.0116–0.0318, and 0.0077–0.0112, respectively. Overall, removing individual components generally reduced the restoration performance.

**Table 13:** Ablation analysis of MSERNet by removing one component at a time under different JPEG degradation conditions. Results are reported as mean ± SD for PSNR, SSIM, and VIF.

JPEG Level	Without multi-scale	Without channel attention	Without residual learning	Without edge refinement	Full MSERNet
	PSNR (dB)				
Q30	38.33 ± 0.96	38.75 ± 0.94	38.32 ± 1.06	38.49 ± 1.00	**39.33 ± 0.96**
Q50	41.39 ± 1.06	40.73 ± 1.03	41.07 ± 1.21	41.57 ± 1.13	**42.48 ± 1.15**
Q70	42.95 ± 1.03	42.35 ± 0.99	42.21 ± 1.24	43.07 ± 1.08	**44.94 ± 1.00**
Q85	46.07 ± 1.25	45.58 ± 1.09	43.86 ± 1.47	46.25 ± 1.36	**47.06 ± 1.19**
Q70_Q30	39.06 ± 0.96	38.54 ± 0.93	39.11 ± 1.05	**39.22 ± 1.00**	39.07 ± 0.96
Q85_Q50	41.37 ± 1.02	40.62 ± 1.01	41.11 ± 1.20	41.57 ± 1.09	**42.73 ± 1.02**
	SSIM				
Q30	0.9396 ± 0.0080	**0.9354 ± 0.0081**	0.9328 ± 0.0079	0.9304 ± 0.0080	**0.9363 ± 0.0048**
Q50	0.9378 ± 0.0049	0.9368 ± 0.0051	0.9454 ± 0.0051	0.9328 ± 0.0050	**0.9499 ± 0.0079**
Q70	0.9545 ± 0.0058	0.9513 ± 0.0060	0.9568 ± 0.0059	0.9552 ± 0.0057	**0.9646 ± 0.0058**
Q85	0.9806 ± 0.0031	0.9894 ± 0.0031	0.9874 ± 0.0035	0.9803 ± 0.0033	**0.9904 ± 0.0030**
Q70_Q30	0.9469 ± 0.0082	0.9429 ± 0.0082	0.9503 ± 0.0082	**0.9477 ± 0.0082**	0.9471 ± 0.0081
Q85_Q50	0.9639 ± 0.0055	0.9602 ± 0.0058	0.9668 ± 0.0054	**0.9648 ± 0.0052**	0.9640 ± 0.0054
	VIF				
Q30	0.5559 ± 0.0719	0.5463 ± 0.0693	0.5544 ± 0.0735	0.5557 ± 0.0743	**0.5667 ± 0.0713**
Q50	0.6460 ± 0.0712	0.6347 ± 0.0691	0.6417 ± 0.0746	0.6467 ± 0.0748	**0.6569 ± 0.0704**
Q70	0.7192 ± 0.0667	0.7095 ± 0.0649	0.7098 ± 0.0717	0.7207 ± 0.0706	**0.7299 ± 0.0656**
Q85	0.7832 ± 0.0561	0.7914 ± 0.0532	0.7708 ± 0.0649	0.7949 ± 0.0607	**0.8026 ± 0.0551**
Q70_Q30	0.5518 ± 0.0729	0.5333 ± 0.0701	0.5411 ± 0.0744	0.5415 ± 0.0751	**0.5527 ± 0.0723**
Q85_Q50	0.6596 ± 0.0675	0.6363 ± 0.0660	0.6470 ± 0.0708	0.6515 ± 0.0710	**0.6603 ± 0.0667**

Bold values highlight the reported results for easier comparison between methods.

### 4.7 Repeated-run and inference evaluation

The repeated-run results are presented in Table 14. MSERNet was trained five times using different random seeds (42, 123, 2024, 7, and 99), while the training, validation, and test split was kept fixed at 382, 82, and 82 images, respectively. The best validation losses obtained across the five runs were 0.006206, 0.005950, 0.005870, 0.006117, and 0.006015, respectively. The mean and standard deviation of the evaluation metrics across the repeated runs are reported to show the variation in model performance. Across the six degradation conditions, PSNR ranged from 39.00 ± 1.01 to 46.03 ± 1.28 dB, SSIM ranged from 0.9373 ± 0.0051 to 0.9904 ± 0.0032, and VIF ranged from 0.5495 ± 0.0729 to 0.8008 ± 0.0564.

**Table 14:** Repeated-run evaluation of MSERNet across five independent training runs. Results are reported as Mean ± Standard deviation (SD) for PSNR, SSIM, and VIF.

Compression type	JPEG level	PSNR (dB)	SSIM	VIF
Single level	Q30	39.26 ± 1.02	0.9492 ± 0.0084	0.5634 ± 0.0721
	Q50	41.33 ± 1.14	0.9373 ± 0.0051	0.6535 ± 0.0717
	Q70	42.90 ± 1.09	0.9643 ± 0.0062	0.7267 ± 0.0672
	Q85	46.03 ± 1.28	0.9904 ± 0.0032	0.8008 ± 0.0564
Recompression	Q70_Q30	39.00 ± 1.01	0.9465 ± 0.0086	0.5495 ± 0.0729
	Q85_Q50	41.29 ± 1.13	0.9636 ± 0.0061	0.6567 ± 0.0685

The computational characteristics of MSERNet are reported in Table 15. FLOPs were calculated as approximately 2 × MACs. All models were evaluated using an input size of 468 × 650 × 1 on an NVIDIA Tesla T4. The computational evaluation showed that MSERNet had fewer parameters, a smaller model size, and lower computational requirements than RRDB and PALMSNet. ARCNN showed lower computational requirements than MSERNet across the evaluated measures.

**Table 15:** Computational complexity and inference efficiency of MSERNet.

Model	Parameters (M)	Model size (MB)	MACs (G)	FLOPs (G)	Inference (ms/image)	GPU memory (MB)
ARCNN (Dong et al., 2015)	0.107	1.23	32.38	64.76	18.35 ± 1.99	229.48
RRDB (Zini et al., 2020)	1.906	22.01	579.27	1,158.55	458.55 ± 4.66	530.02
PALMSNet (Jain et al., 2026)	7.417	84.98	508.04	1,016.08	394.36 ± 4.01	1,152.22
MSERNet	0.467	1.78	141.17	282.34	71.20 ± 0.689	602.45

M, million; MB, megabytes; G, giga; ms, milliseconds; MACs, multiply–accumulate operations; FLOPs, floating-point operations.

### 4.8 Downstream classification evaluation

To further assess whether JPEG artifact restoration preserves information relevant to a subsequent medical image-analysis task, a downstream binary classification experiment was conducted using the MSERNet-restored images. A transfer learning–based ResNet50 classifier pretrained on ImageNet was used to classify the images into cystic and normal classes. The same test set and classification procedure were used for the restored images obtained under the different JPEG degradation conditions. While PSNR, SSIM, and VIF were used to assess restoration quality, downstream classification performance was evaluated using accuracy, precision, sensitivity, specificity, F1-score, and AUC. The classification results are summarized in Table 16, and the corresponding confusion matrices are presented in Figure 9. A transfer learning–based ResNet50 classifier pretrained on ImageNet was evaluated on the same 53-image test set across all MSERNet restoration conditions. The best checkpoint was obtained at Epoch 14 with a validation loss of 0.005926. MSERNet-Q30 and MSERNet-Q70_Q30 achieved an accuracy of 0.9434, sensitivity of 0.9231, specificity of 0.9630, and F1-score of 0.9412, whereas the remaining conditions achieved 0.9623 accuracy, 0.9615 sensitivity, 0.9630 specificity, and 0.9615 F1-score. AUC values were 0.9943 for all conditions except Q70_Q30 (0.9929).

**Table 16:** Downstream binary classification performance of the transfer learning–based ResNet50 classifier on MSERNet-restored ovarian ultrasound images under different JPEG degradation conditions.

Restoration condition	Accuracy	Sensitivity	Specificity	F1-score	AUC
MSERNet-Q30	96.23%	92.31%	96.30%	94.12%	99.43%
MSERNet-Q50	96.23%	96.15%	96.30%	96.15%	99.43%
MSERNet-Q70	96.23%	96.15%	96.30%	96.15%	99.43%
MSERNet-Q85	96.23%	96.15%	96.30%	96.15%	99.43%
MSERNet-Q70_Q30	94.34%	92.31%	96.30%	94.12%	99.29%
MSERNet-Q85_Q50	96.23%	96.15%	96.30%	96.15%	99.43%

**Figure 9:**
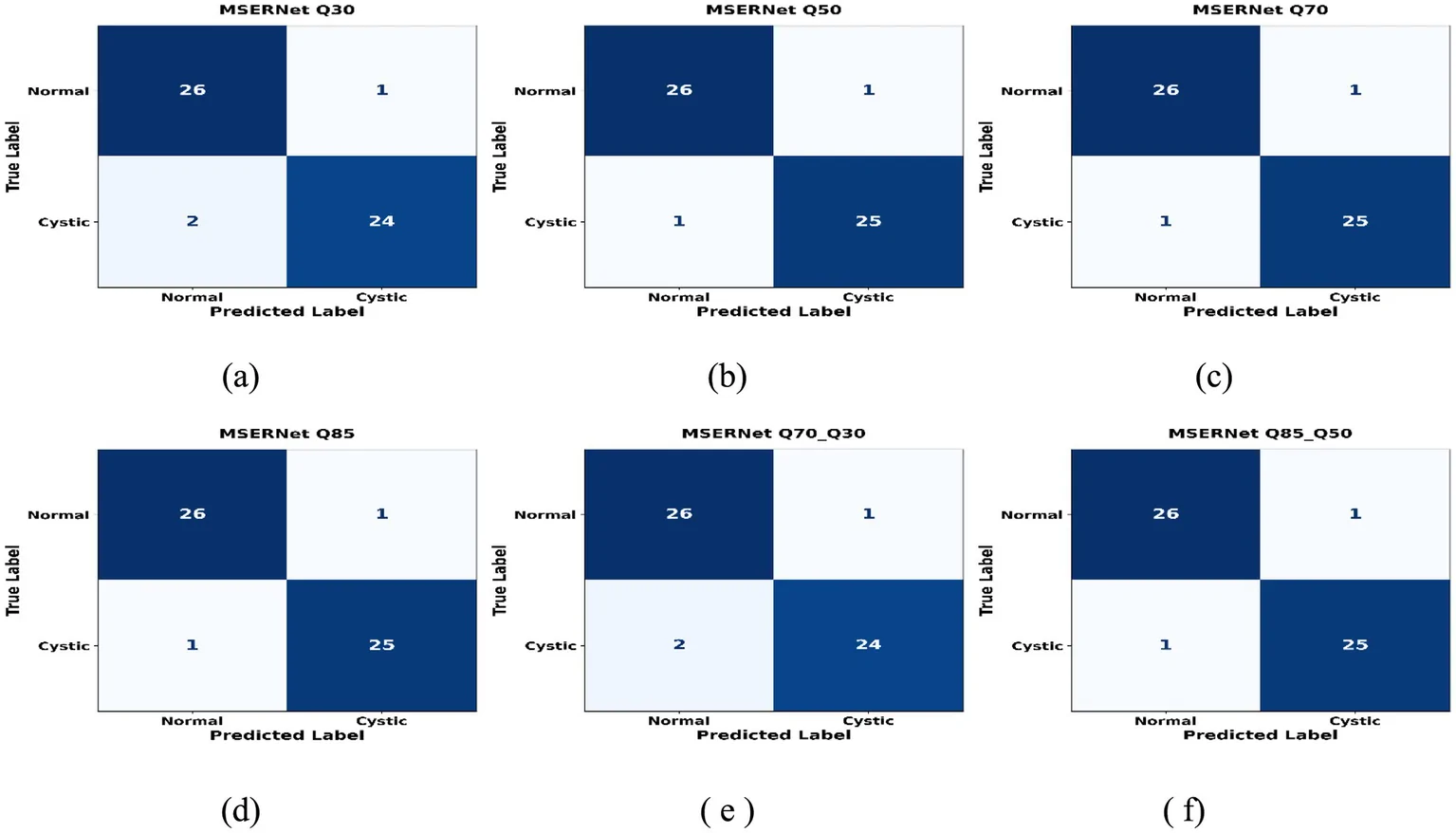
Confusion matrices for the transfer learning–based ResNet50 classifier evaluated on MSERNet-restored ovarian ultrasound images: **(a)** Q30, **(b)** Q50, **(c)** Q70, **(d)** Q85, **(e)** Q70_Q30, and **(f)** Q85_Q50.

### 4.9 Statistical validation of cross-domain performance (paired *t*-test)

To further validate the cross-domain performance of the proposed MSERNet, a paired *t*-test was performed using the 15% independent public test set (82 paired images). The analysis compared the restoration performance of the model trained and tested on the public dataset with the cross-domain model trained on the private dataset and evaluated on the same public test images. Image-wise PSNR, SSIM, and VIF values were analysed separately for each JPEG compression and recompression level. Statistical significance was evaluated at α = 0.05. The statistical results are summarized in Table 17. The public-domain evaluation showed higher mean performance than the cross-domain evaluation across the evaluated conditions. The observed differences (Public − CD) ranged from 0.40 to 1.68 dB for PSNR, 0.00 to 0.06 for SSIM, and 0.00 to 0.02 for VIF. All comparisons were statistically significant (*p* < 0.001). These results indicate measurable differences between the public-domain and cross-domain evaluations and provide an independent assessment of MSERNet’s cross-dataset robustness on the evaluated ultrasound datasets. Statistical significance was not interpreted as evidence of clinical equivalence.

**Table 17:** Paired *t*-test comparison of restoration performance between the public and cross-domain datasets across different compression levels.

JPEG level	Public			Cross-domain (CD)			Δ (Public – CD)			*t*-value			*p*-value	Significant
	PSNR	SSIM	VIF	PSNR	SSIM	VIF	PSNR	SSIM	VIF	PSNR	SSIM	VIF		
Q30	42.12	0.98	0.72	40.86	0.92	0.70	1.26	0.06	0.02	47.25	40.37	85.39	<0.001	Yes
Q50	44.69	0.99	0.80	43.09	0.93	0.78	1.60	0.06	0.02	40.24	37.75	84.79	<0.001	Yes
Q70	46.90	0.99	0.85	46.05	0.99	0.83	0.85	0.00	0.02	59.27	38.58	65.72	<0.001	Yes
Q85	48.93	1.00	0.89	48.53	1.00	0.89	0.40	0.00	0.00	19.53	20.76	20.04	<0.001	Yes
Q70_Q30	41.78	0.97	0.71	40.61	0.91	0.69	1.17	0.06	0.02	47.49	39.44	82.07	<0.001	Yes
Q85_Q50	44.72	0.99	0.80	43.04	0.93	0.78	1.68	0.06	0.02	41.73	38.66	86.18	<0.001	Yes

Δ = Public − CD. Positive Δ values indicate higher performance for the public-domain evaluation. The t-values are reported separately for PSNR, SSIM, and VIF. Statistical significance was evaluated at *α* = 0.05; all comparisons were statistically significant (*p* < 0.001).

## 5 Discussion

### 5.1 Restoration performance across different compression levels

The quantitative results in Table 11 demonstrate that the proposed MSERNet consistently restores JPEG-compressed ultrasound images across different compression levels and recompression scenarios. Although restoration performance gradually improves from Q30 to Q85 due to the greater structural information retained at higher quality factors, the most important finding is the stable performance achieved under severe compression (Q30 and Q50) and recompression conditions (Q70_Q30 and Q85_Q50). These results show that MSERNet performs consistently across different levels of JPEG degradation. The multi-scale feature extraction, adaptive feature fusion, and edge refinement help reduce compression artifacts. These components also help preserve tissue textures and anatomical boundaries.

### 5.2 Comparison with existing methods

The comparison methods were selected to represent different image restoration approaches, including conventional medical ultrasound restoration (Ahemed et al., 2025), deep CNN-based methods (Zini et al., 2020; Dong et al., 2015), a recent CNN-based method (Jain et al., 2026), and a DCT-domain Transformer-based method (Ouyang and Chen, 2024). All methods were evaluated on the same ovarian ultrasound dataset using identical JPEG degradation levels and evaluation metrics. As shown in Table 12, the restoration results varied across JPEG degradation levels and evaluation metrics. The DCT-based Transformer (Ouyang and Chen, 2024) achieved higher PSNR values at Q30 and Q70_Q30, whereas MSERNet achieved higher PSNR values at Q50, Q70, Q85, and Q85_Q50. Differences were also observed for SSIM and VIF, with PALMSNet (Jain et al., 2026) and Wiener filtering (Ahemed et al., 2025) achieving higher values under some conditions.

These differences may be related to the restoration strategies used by the compared methods. Wiener filtering relies on predefined image-processing operations (Ahemed et al., 2025), while ARCNN (Dong et al., 2015), RRDB (Zini et al., 2020), PALMSNet (Jain et al., 2026), and the DCT-based Transformer (Ouyang and Chen, 2024) use learned or frequency-domain approaches. MSERNet combines multi-scale feature extraction, channel attention, edge refinement, and residual learning to address JPEG artifacts while preserving structural details in ovarian ultrasound images.

Overall, Table 12 shows that no single method achieved the highest values across all metrics and degradation levels. The results indicate that restoration performance depends on the compression condition and the characteristics of the restoration method. These components may help the network capture different image structures and local variations in ovarian ultrasound images. Overall, the comparison shows that restoration behavior depends on both the degradation level and the restoration approach, highlighting the importance of evaluating JPEG artifact restoration within the target ultrasound domain.

### 5.3 Ablation study analysis

The ablation results indicate that the four components contribute differently to the restoration process in Table 13. Multi-scale processing allows information from different image scales to be used, while channel attention helps the network focus on useful feature responses. Residual learning supports the recovery of information lost during JPEG degradation, and edge refinement helps preserve local image details. The reduction in VIF after removing each component under all tested conditions suggests that the combined components help retain image information during restoration The variations observed in PSNR and SSIM also show that the contribution of an individual component can change with the degradation. Therefore, the results support the use of the complete MSERNet configuration for JPEG artifact restoration in ovarian ultrasound images.

### 5.4 Repeated-run and inference evaluation

The repeated-run evaluation was performed to assess the stability of MSERNet across different training runs (Table 14). Five runs were conducted using different random seeds while keeping the training, validation, and test split unchanged. The reported mean ± standard deviation values show the variation in restoration performance across these runs. The relatively small variation in PSNR, SSIM, and VIF indicates that the measured performance was not dependent on a single training run.

The computational requirements should also be considered in the context of the target medical imaging application. The proposed MSERNet was developed and evaluated on a private ovarian ultrasound dataset, where restoration is performed on relatively high-resolution single-channel images. The network combines multi-scale processing, channel attention, residual learning, and edge refinement to address different aspects of JPEG degradation in these images. These components introduce additional computational operations compared with simpler networks such as ARCNN (Dong et al., 2015). However, the measured results show that MSERNet requires fewer parameters, a smaller model size, and lower computational cost than the larger RRDB (Zini et al., 2020) and PALMSNet (Jain et al., 2026) models under the same evaluation setting (Table 15) Thus, the computational measurements provide a dataset-specific assessment of the resources required for applying MSERNet to the ovarian ultrasound restoration task.

### 5.5 Downstream classification evaluation

The confusion matrices and classification metrics show that the MSERNet-restored images retained information sufficient for the evaluated cystic-versus-normal classification task (Table 16; Figure 9). The Q50, Q70, Q85, Q95, and Q85_Q50 conditions produced identical results, with 51 of 53 test images correctly classified, including 25 of 26 cystic and 26 of 27 normal images (Figure 9). In contrast, Q30 and Q70_Q30 produced 50 correct predictions, with two cystic images misclassified as normal (Figure 9). This resulted in lower sensitivity (0.9231) and F1-score (0.9412), while specificity remained 0.9630 across all conditions. Overall, the similar classification performance across most restoration conditions indicates that the restored images retained information that supported the evaluated downstream classification task. However, this evaluation used a 53-image test set and does not establish improved clinical diagnostic performance.

### 5.6 Qualitative analysis of the proposed MSERNet

The qualitative comparisons in Figures 6–8 are consistent with the quantitative evaluation. For the private ovarian ultrasound dataset, the restored images show reduced blocking artifacts while retaining tissue textures and anatomical boundaries. A similar pattern is observed in the public carotid ultrasound dataset, where vessel boundaries and surrounding tissue structures are preserved at different compression levels. The enlarged ROI provides a closer view of subtle anatomical structures and shows reduced compression artifacts without evident over-smoothing. The consistent visual quality observed in the cross-dataset experiments provides evidence of the cross-dataset robustness of MSERNet across the two evaluated datasets.

### 5.7 Generalization across private, public, and cross domain dataset evaluation

The private ovarian ultrasound dataset contains greater variation in patient anatomy, imaging protocols, and scanner settings than the public dataset. This variation makes the restoration task more challenging. In comparison, the public dataset has more consistent image characteristics and acquisition conditions. Despite these differences, MSERNet achieved comparable restoration performance on both datasets based on PSNR, SSIM, and VIF. The multi-scale feature extraction, adaptive feature fusion, and edge refinement modules are designed to reduce compression artifacts while retaining structural details. The cross-dataset evaluation also produced results close to those obtained without additional fine-tuning. As shown in Figures 6–8 and Table 11, the quantitative and qualitative comparisons demonstrate that MSERNet maintained similar restoration performance under the different imaging conditions represented by the two datasets. The improvements in PSNR, SSIM, and VIF indicate better image fidelity, structural similarity, and information preservation after restoration. However, these metrics alone cannot confirm an improvement in diagnostic performance. The downstream classification results presented in Table 16 provide additional evidence that relevant image information is preserved in the MSERNet-restored images.

## 6 Conclusion

This proposed work MSERNet, a multi-scale network for restoring JPEG-compressed ultrasound images. A curated private ovarian ultrasound dataset comprising ethically approved anonymized clinical images with controlled compression and recompression levels was developed to provide a systematic evaluation framework. By integrating multi-scale feature extraction, adaptive feature fusion, and edge refinement, the proposed network effectively reduced JPEG compression artifacts while preserving important anatomical structures. The proposed framework was validated on both private and public ultrasound datasets, together with cross-dataset evaluation, demonstrating stable restoration performance under different compression conditions. Comparative evaluation with existing restoration methods, ablation analysis, repeated-run evaluation, statistical analysis, and computational analysis were also performed to assess restoration performance, component contributions, consistency, and computational characteristics. The consistent quantitative improvements and qualitative restoration results demonstrate that MSERNet provides an effective restoration framework for JPEG-compressed ultrasound images across the evaluated datasets and compression conditions. A downstream binary classification experiment using a transfer learning-based ResNet50 classifier further demonstrated that the restored images retained information relevant to cystic-versus-normal classification based on accuracy, precision, sensitivity, specificity, F1-score, and AUC. The proposed framework can therefore support subsequent medical image analysis by improving the quality of compressed ultrasound images.

### 6.1 Limitations and future work

The proposed MSERNet was evaluated using grayscale ultrasound images from private and public datasets under JPEG compression. Other imaging modalities and compression standards were not included. Although downstream binary classification was performed, the evaluation was limited to the available datasets and does not directly establish improved clinical diagnostic performance. Future work will evaluate MSERNet on larger and more diverse datasets, additional compression schemes, and other medical image-analysis applications, including lesion segmentation, detection, and classification.

## Data Availability

The data generated and/or analysed during the present study are not publicly available due to hospital privacy policies and data confidentiality requirements. The private ovarian ultrasound dataset can only be accessed by authorized personnel. The public Common Carotid Artery ultrasound dataset used in this study is available from Mendeley Data DOI: 10.17632/d4xt63mgjm.1.
